# Impact of the COVID-19 crisis on work and private life, mental well-being and self-rated health in German and Swiss employees: a cross-sectional online survey

**DOI:** 10.1186/s12889-021-10788-8

**Published:** 2021-04-17

**Authors:** Martin Tušl, Rebecca Brauchli, Philipp Kerksieck, Georg Friedrich Bauer

**Affiliations:** Public & Organizational Health, Center of Salutogenesis, Epidemiology, Biostatistics and Prevention Institute, University of Zurich, Hirschengraben 84, 8001 Zurich, Switzerland

**Keywords:** COVID-19, Online survey, Logistic regression, Work life, Private life, Mental well-being, Self-rated health, Germany, Switzerland

## Abstract

**Background:**

The COVID-19 crisis has radically changed the way people live and work. While most studies have focused on prevailing negative consequences, potential positive shifts in everyday life have received less attention. Thus, we examined the actual and perceived overall impact of the COVID-19 crisis on work and private life, and the consequences for mental well-being (MWB), and self-rated health (SRH) in German and Swiss employees.

**Methods:**

Cross-sectional data were collected via an online questionnaire from 2118 German and Swiss employees recruited through an online panel service (18–65 years, working at least 20 h/week, various occupations). The sample provides a good representation of the working population in both countries. Using logistic regression, we analyzed how sociodemographic factors and self-reported changes in work and private life routines were associated with participants’ perceived overall impact of the COVID-19 crisis on work and private life. Moreover, we explored how the perceived impact and self-reported changes were associated with MWB and SRH.

**Results:**

About 30% of employees reported that their work and private life had worsened, whereas about 10% reported improvements in work and 13% in private life. Mandatory short-time work was strongly associated with perceived negative impact on work life, while work from home, particularly if experienced for the first time, was strongly associated with a perceived positive impact on work life. Concerning private life, younger age, living alone, reduction in leisure time, and changes in quantity of caring duties were strongly associated with perceived negative impact. In contrast, living with a partner or family, short-time work, and increases in leisure time and caring duties were associated with perceived positive impact on private life. Perceived negative impact of the crisis on work and private life and mandatory short-time work were associated with lower MWB and SRH. Moreover, perceived positive impact on private life and an increase in leisure time were associated with higher MWB.

**Conclusion:**

The results of this study show the differential impact of the COVID-19 crisis on people’s work and private life as well as the consequences for MWB and SRH. This may inform target groups and situation-specific interventions to ameliorate the crisis.

**Supplementary Information:**

The online version contains supplementary material available at 10.1186/s12889-021-10788-8.

## Key findings

- 31% of employees perceived a negative impact of the crisis on their work life. Mandatory short-time workers and those who lost their job felt the negative impact the most.
- 10% of employees perceived a positive impact of the crisis on their work life. Those working in home-office, particularly if experienced for the first time, felt the positive impact the most.
- 30% of employees perceived a negative impact of the crisis on their private life. Living in a single household, reduction in leisure time, and changes in quantity of caring duties (i.e., increase or decrease) were strongly associated with the negative impact.
- 13% of employees perceived a positive impact on their private life. Living with a partner or family, mandatory short-time work, increases in leisure time and caring duties were strongly associated with the positive impact.
- Perceived negative impact of the crisis on work and private life and mandatory short-time work were strongly associated with lower mental well-being and self-rated health.
- Perceived positive impact of the crisis on private life and an increase in leisure time were strongly associated with higher mental well-being and, for leisure time, also with higher self-rated health.
- Targeted interventions for vulnerable groups should be established on a company/governmental levels such as psychological first aid accessible online or rapid financial aids for those who have lost their income partially or completely.
- Companies may consider offering positive psychology trainings to employees to help them purposefully focus on and make use of the beneficial consequences of the crisis. Such trainings may also include workshops on optimal crafting of their work and leisure time during the pandemic.

## Background

On January 30, 2020, the World Health Organization (WHO) declared the outbreak of COVID-19 a Public Health Emergency of International Concern (PHEIC) [1]. In the following weeks, the virus quickly spread worldwide, forcing the governments of affected countries to implement lockdown measures to decrease transmission rates and prevent the overload of hospital emergency rooms. Switzerland entered full lockdown on March 16th, Germany followed 6 days later on March 22nd. Restrictive measures in both countries were comparable and included border controls, closing of schools, markets, restaurants, nonessential shops, bars, entertainment and leisure facilities, as well as ban on all public and private events and gatherings [2, 3]. Such strict measures were in place until the end of April when both governments started to gradually ease the measures [4, 5]. Consequently, much of the working population suddenly faced drastic changes to everyday life. People who commuted to work and had rich social lives outside their homes found themselves in a mandatory work from home (WFH) situation, many employees were furloughed or laid off as various businesses and industries had to shut down, and health workers in emergency rooms as well as supermarket staff and other essential employees were faced with a dramatic increase in workload and job strain [6, 7].

Regarding the public health impact of the COVID-19 crisis, several studies suggest that working conditions have deteriorated and that employees are more likely to experience mental health problems, such as stress, depression, and anxiety [8–11]. In particular, women, young adults, people with chronic diseases, and those who have lost their jobs as a result of the crisis seem to be the most affected [11–14]. One of the common stressors that research has highlighted is the fear of losing one’s job and, consequently, one’s income [7]. Moreover, social isolation, conflicting messages from authorities, and an ongoing state of uncertainty have been described as some of the main factors contributing to emotional distress and negatively affecting mental health and well-being [8, 14–18].

In the European context, Eurofound [12] released a report on research in April 2020 involving 85,000 participants across 27 EU member countries. The data indicate that the EU population experienced high levels of loneliness, low levels of optimism, insecurity regarding their jobs and financial future, as well as a decrease in well-being. Germany scored slightly below the EU27 average in well-being, and there is further evidence that it decreased significantly in the early stages of the COVID-19 pandemic, between March 2020 and May 2020 [19]. The Eurofound report does not discuss Switzerland; however, other studies suggest that there has been an increase in emotional distress in Swiss young adults [20] and that undergraduate students have experienced higher levels of stress, depression, anxiety, and loneliness compared to the time before the COVID-19 outbreak [14]. A Swiss social monitor study reports that over 40% of Swiss adults perceive a worsened quality of life compared to before the pandemic, 10% experience feelings of loneliness, 10% report fear of losing their job, and about 1% lost their job as a result of the pandemic. The report also indicates an increase in WFH by 29% compared to before the pandemic [21].

Accordingly, the data from Eurofound [12] also suggest that European employees have experienced a dramatic increase in WFH. About 37% of the EU working population transitioned to WFH as a result of the pandemic, and 24% WFH for the first time. Before the pandemic, employees had considered remote working a benefit when it followed their preferences. However, the COVID-19 lockdown changed this by forcing many employees into mandatory WFH [6]. This posed various challenges for employees without prior WFH experience, such as organizing the workspace, establishing new communication channels with colleagues, coping with work isolation, or managing boundaries between work and non-work [22–24]. Without proper support from the employer or insufficient resources to manage these challenges, mandatory WFH may become a burden that negatively affects employees’ well-being [8] and, in turn, their performance [22]. Furthermore, the increase in WFH has been highlighted as a potential threat to parents with small children at home, as this group is likely to experience difficulties in combining work duties with home schooling and household chores [12, 23].

Indisputably, the COVID-19 pandemic has had a strong impact on many aspects of our lives and will continue to do so for months and years to come. However, the consequences of the crisis and societal reactions to the challenges posed by the virus are not deemed solely negative. The new situation also holds opportunities for positive shifts in our work and private lives that were impossible before the COVID-19 crisis. Many may see this crisis as an opportunity to learn how to cope with profound changes in everyday life and even to adopt new pro-active behaviors. For instance, some employees may discover that the new ways of working (e.g., WFH) facilitate more productivity and are more satisfying compared to working in an office [25]. Data collected from employees in Denmark and Germany between March and May 2020 [26] suggest that 71% of respondents felt informed and well prepared for the changing work situation and WFH. Participants also reported several advantages of working from home, such as perceived control over the workday, working more efficiently, or saving time previously spent commuting. In contrast, some reported disadvantages of WFH included social isolation, loss of the value of work, and a lack of important work equipment. Nonetheless, respondents reported overall relatively more positive experiences of WFH than negative ones. Thus, we argue that more balanced studies are needed that examine both the negative and positive impact of the COVID-19 crisis on peoples’ lives, health, and well-being, considering differential effects in diverse subgroups. Such studies have the potential to conclude how to diminish the negative and enhance the positive outcomes of the current and future pandemic-related crises in the working population.

### Aim and objectives

The overall aim of the present study was to examine the actual and perceived overall impact of the COVID-19 crisis on employees’ work and private life, along with its consequences for mental well-being (MWB) and self-rated health (SRH) in the German and Swiss working populations. Specifically, we pursued the following objectives:

1. To investigate the perceived positive and negative impact of the COVID-19 crisis on work and private life as well as to assess the self-reported changes in work and private life routines induced by the crisis.
2. To examine which sociodemographic variables and which self-reported changes in work and private life routines are associated with perceived positive and negative impact of the COVID-19 crisis on work and private life.
3. To investigate how the self-reported changes and perceived overall impact of the COVID-19 crisis on work and private life are associated with MWB and SRH as relevant health outcomes.

Although SRH has been identified as a relevant predictor of mental distress during the COVID-19 pandemic [10, 27], to our knowledge, it has not been studied as an outcome variable in combination with MWB indicators as in our study.

## Methods

The present study used a cross-sectional online survey design. We report our study following the STROBE guidelines for cross-sectional studies [28], and the checklist for reporting results of internet e-surveys (CHERRIES) [29], see ‘Additional file 1.pdf’ in supplementary material.

### Sample

Participants were recruited through a panel data service Respondi (respondi.com). Cross-sectional data were collected from employees in Germany and Switzerland via an online questionnaire using a web-based survey provider SurveyGizmo. The questionnaire was tested and checked by senior researchers from the field for face validity prior to the administration. The period of data collection was from 9th to 22nd April 2020, when both countries were in full lockdown as part of the control measures relating to COVID-19. Participants received a minimal incentive for completing the survey (i.e., points which could be redeemed towards a given service after participating in several surveys). Participation was voluntary and participant anonymity and confidentiality of their data were assured and emphasized. Each participant in the online panel service database had a unique code which ensured anonymity and prevented multiple submissions from one participant. Important items in the survey were mandatory and participants were informed if they accidently skipped an item. Further, the questionnaire used a logic to avoid asking redundant or non-applicable questions (e.g., participants who indicated that they lost their job were not asked about the change in working time or home-office). Moreover, we included several disqualifying items (i.e., “Please choose number three as an answer to this item”) as a quality check to exclude participants who would give random answers. Participants were able to go back in the survey and review or change their answers.

**Fig. 1:**
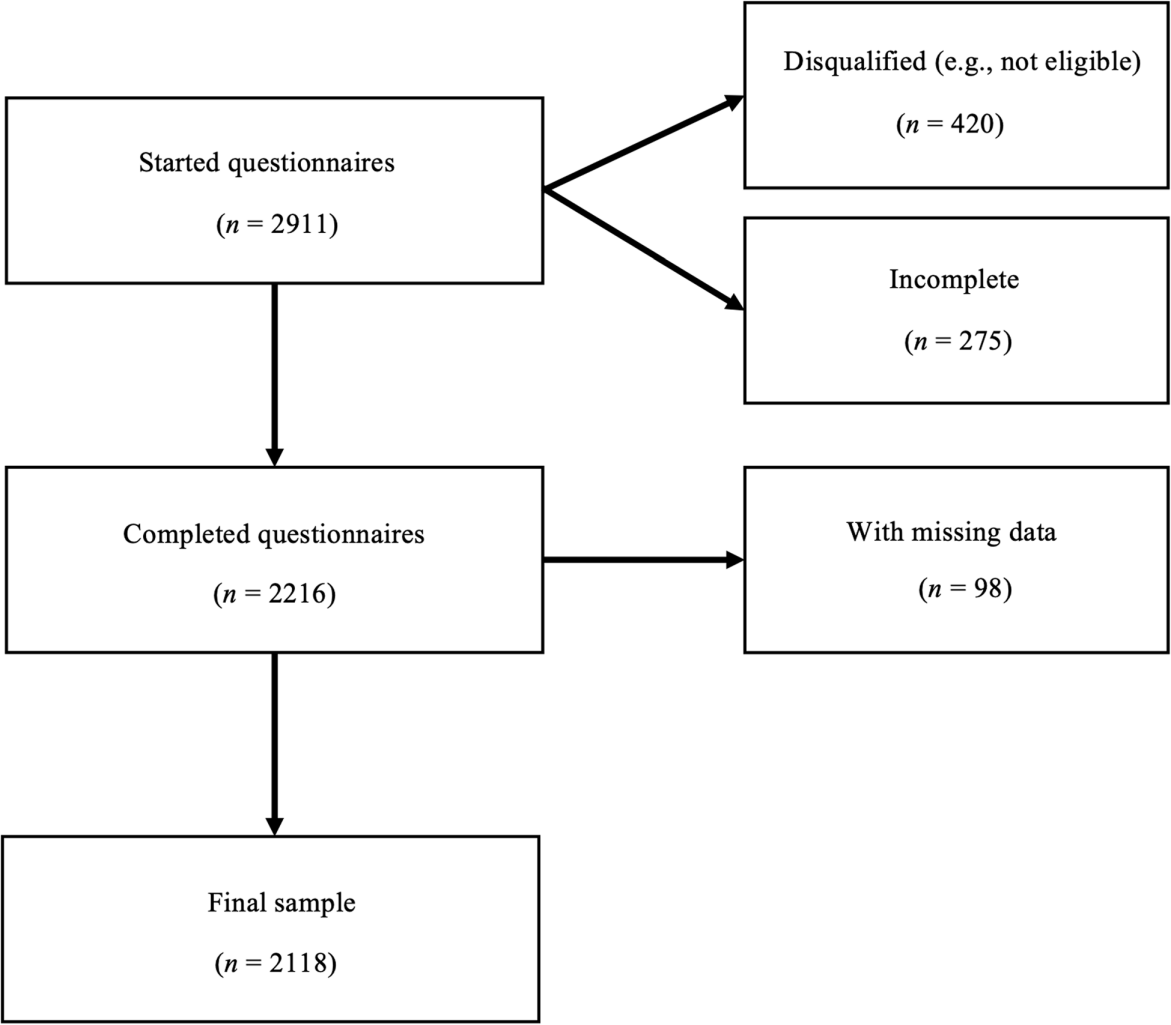
Sample flow diagram

The eligibility criteria were: being employed (not self-employed), working more than 20 h per week, and being within the age range of 18 to 65 years. The final sample included 2118 participants. Figure 1 shows a flow diagram describing how the final sample was achieved.

Sociodemographic characteristics of the sample are shown in Table 1: the mean age was 46.51 years (*SD* = 11.28), 5% completed primary, 58% secondary, and 37% tertiary education,^1^ 55% were male, 77% were from Germany, and 72% were living with a partner, family, or in a shared housing.

**Table 1:** Sociodemographic characteristics of the sample

	*N*	(%)
Gender		
Male	1160	55
Female	958	45
Country		
Germany	1629	77
Switzerland	489	23
Age		
18–30	208	10
31–40	481	23
41–50	533	25
51–60	675	32
61–65	221	10
Living situation		
Alone	587	28
Family/partner/shared	1531	72

Note: *N* = 2118

Overall, in terms of age, education, and living situation (i.e., single households), the study sample seems to be a good representation of the target of the working population in Germany (www.destatis.de) and Switzerland (www.bfs.admin.ch). In general, males were slightly overrepresented in our sample (56%) compared to the general population (52%); however, the proportion of males in both countries did not differ significantly (56% from Germany, 52% from Switzerland), *χ*^2^(1) = 1.63, *p* = 0.201.

### Measures

#### Perceived overall impact of COVID-19 on work and private life

Assuming that both improvements and deteriorations can simultaneously occur due to COVID-19, we designed four separate items (see ‘Additional file 2.pdf’ in supplementary material) to assess participants’ subjective evaluation of the overall impact of the COVID-19 crisis on their work and private lives: “The Corona-crisis has (a) worsened my work life; (b) improved my work life; (c) worsened my private life; (d) improved my private life.” The response scale ranged from 1 = *strongly disagree* to 5 = *strongly agree*. As a primer to this question, we defined the Corona-crisis as follows:

“The following questions deal directly with the current COVID-19 (Corona) pandemic and the consequent regulations from the government (i.e., business closures, school closures, event bans, contact reduction in public spaces, etc.). Hereafter, we refer to this collectively as the Corona-crisis. Please compare your current situation with the situation as it was before the government regulations.”

#### Changes in work and private life routines

The following items examined qualitative and quantitative changes in participants’ work and private life routines resulting from the COVID-19 crisis: (a) change in employment contract (*no change*; *short-time work*^2^*with a reduced contract*; *short-time work with a contract reduced to 0 h*; *job loss*); (b) proportion of WFH before and after COVID-19 (*0* to *100%*; participants were grouped into three categories according to their answers: *None*, *Experienced*, *New*^3^); (c) changes in quantity of working time,; (d) changes in quantity of leisure time; and (e) changes in quantity of caring duties. The response scale for items c, d, and e ranged from 1 = *strongly decreased* to 5 = *strongly increased*. For the statistical analysis, responses were grouped into three categories: *decreased* (1 + 2), *unchanged* (3), *increased* (4 + 5).

#### Mental well-being

MWB was assessed with the Warwick-Edinburgh Mental Well-Being Scale (WEMWBS) [30]. Specifically, we used the German translation of the 7-item short version of the WEMWBS [31]. WEMWBS is a measure of MWB capturing the positive aspects of mental health, namely, positive affect (feelings of optimism, relaxation), satisfying interpersonal relationships, and positive functioning (clear thinking, self-acceptance, competence, autonomy). The response scale ranged from 1 = *never* to 5 = *all the time*. For the statistical analysis (i.e., ordinal logistic regression model), we grouped participants into six categories according to their overall score in percentiles (10, 25, 50, 75, 90, 99%).

#### Self-rated health

SRH was assessed with a single item: “In general, how would you evaluate your health?” [32]. The response scale ranged from 1 = *very bad* to 5 = *very good*. The application of single-item measures for self-evaluated health is a gold standard in public health research [33].

### Statistical analysis

Data analysis was carried out using R version 4.0.2. In the first step, four ordinal logistic regression models using *polr* from the *MASS* R package [34] were fitted to assess associations of the perceived overall impact of COVID-19 on work and private life as outcome variables with sociodemographic factors (gender, age, country, living situation) and factors related to changes in work and private life routines (changes in employment contract, WFH, work time, leisure time, caring duties) as independent variables. To verify that there was no multicollinearity, the variables were tested a priori using the variance inflation factor tested *vif* from the *car* R package [35] (VIF < 2). The results are presented as adjusted odds ratio (OR) with 95% confidence intervals (95% CI) interpreted as the OR of reporting a higher level of the impact compared to the reference category.

Further, two additional ordinal logistic regression models were fitted to investigate the association between the perceived overall impact of COVID-19 on work and private life^4^ and the self-reported changes in work and private life routines as independent variables and MWB with SRH as outcome variables. In both models, we also controlled for possible confounders (gender, age, country, living situation). The results are presented as adjusted OR with 95% CI interpreted as the OR of reporting a higher level of MWB/SRH compared to the reference category.

## Results

**Fig. 2:**
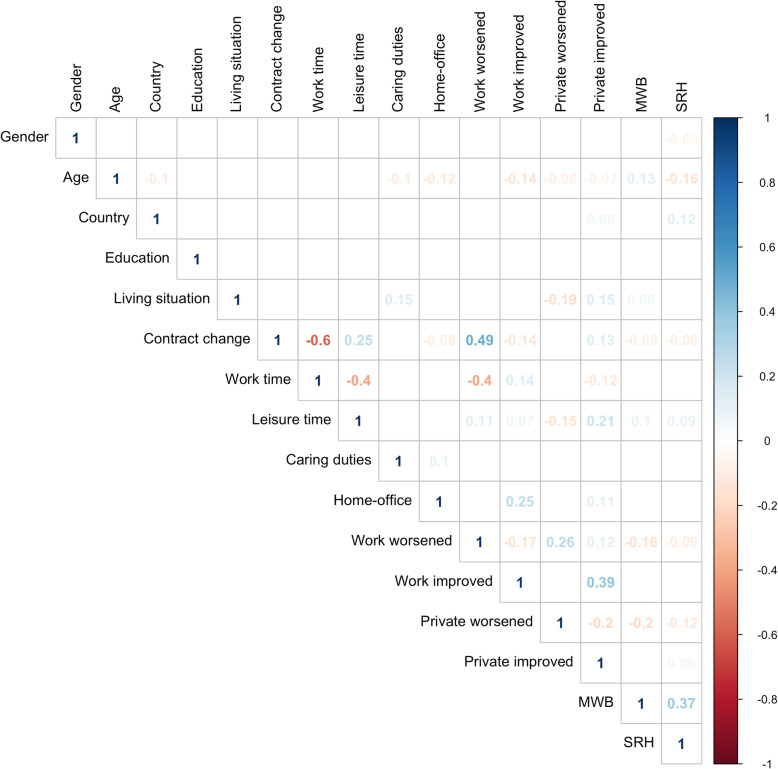
Correlation matrix of the analyzed variables. Note: Only correlations with *p* < 0.01 displayed; Gender (1 = Female, 2 = Male); Country (1 = Germany, 2 = Switzerland); Education (1 = Primary, 2 = Secondary, 3 = Tertiary); Living situation (1 = Alone, 2 = With partner/family); Contract change (1 = No change, 2 = Short-time reduced, 3 = Short-time 0, 4 = Job loss); Home-office (1 = None, 2 = Experienced, 3 = New)

Figure 2 displays the correlations between the analyzed variables. Education was not included in the regression models due to missing data (see details in the Methods section).

### Perceived overall impact of COVID-19 crisis and self-reported changes in work and private life routines

**Fig. 3:**
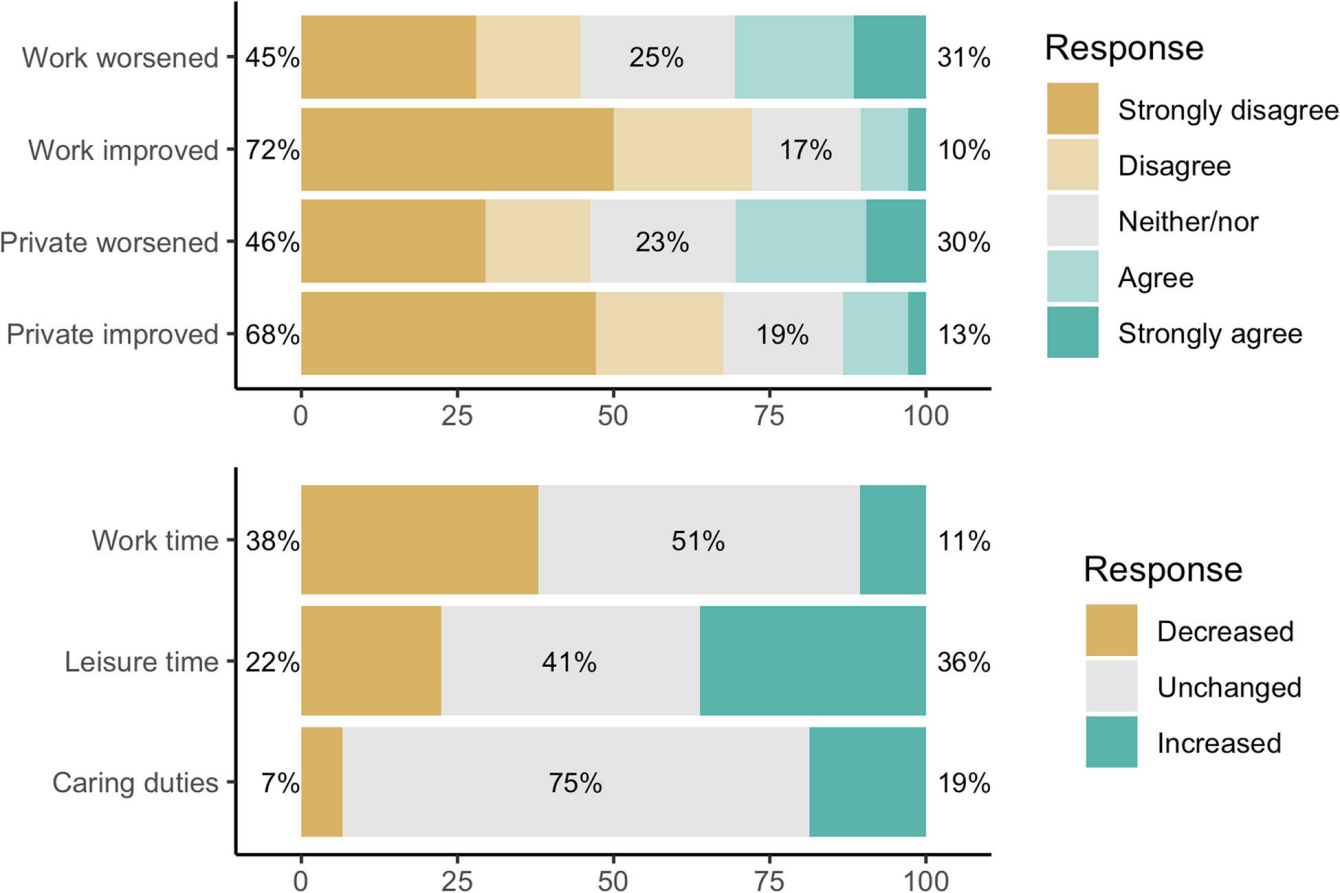
Perceived impact on work and private life and self-reported changes in work time, leisure time, and caring duties. Note: Total percentage does not always equal 100% due to rounding error

Figure 3 shows the results for the four items related to the perceived overall impact of the COVID-19 crisis on work and private life. Thirty-one percent of participants (strongly) agreed that their work life had worsened and 30% (strongly) agreed that their private life had worsened. In contrast, 10% (strongly) agreed that their work life had improved and 13% (strongly) agreed that their private life had improved as a result of the COVID-19 crisis.

Further, Fig. 3 shows self-reported changes with regard to the quantity of time actually spent in work and private life. Work time decreased for 38%, leisure time increased for 36%, while the amount of caring duties changed for 26% of participants.

**Fig. 4:**
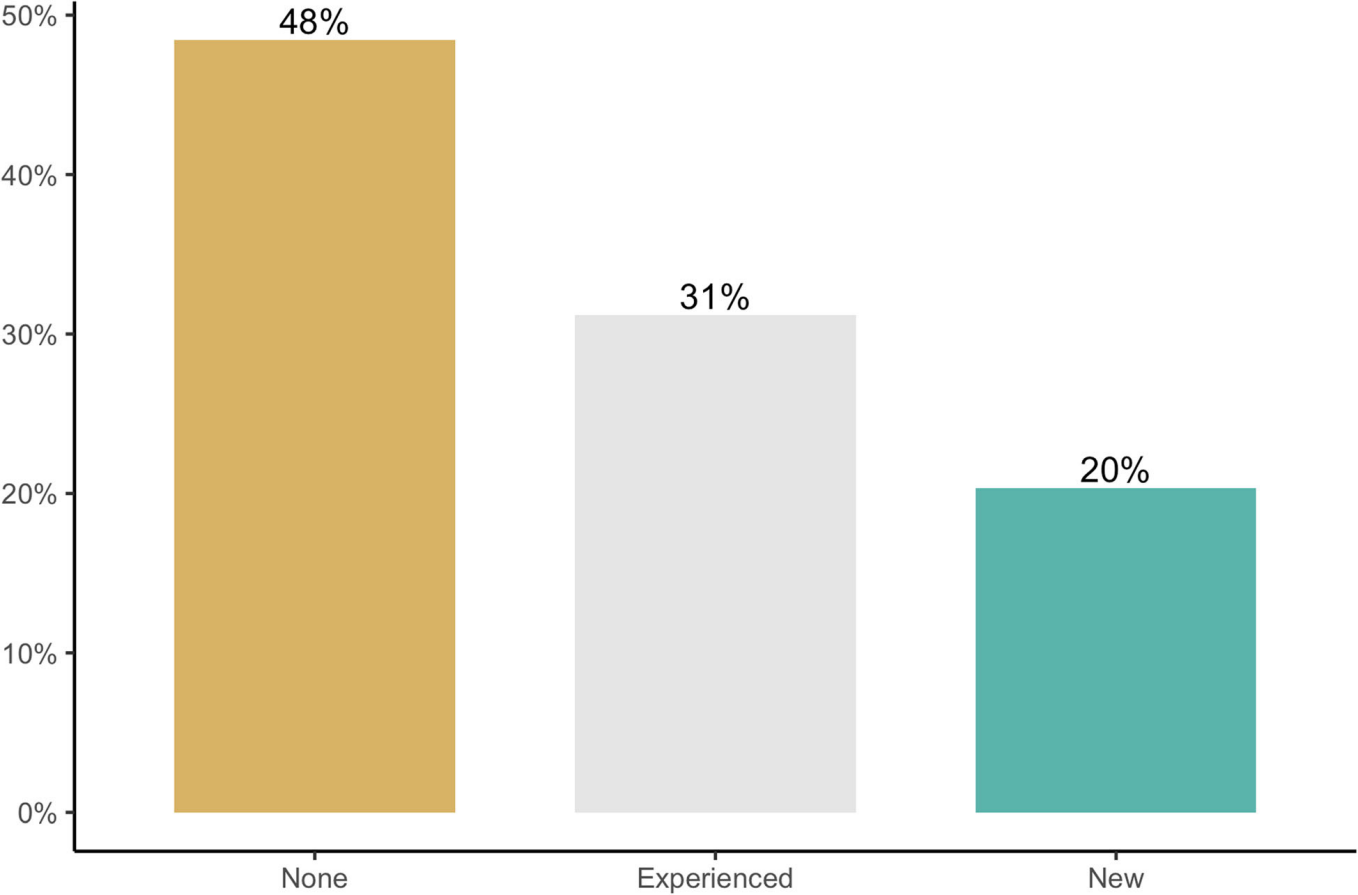
Self-reported changes in home-office. Note: None = 0% WFH before COVID-19, 0% after; Experienced = at least 10% WFH before and at least 10% after COVID-19; New = 0% WFH before and at least 10% after COVID-19

**Fig. 5:**
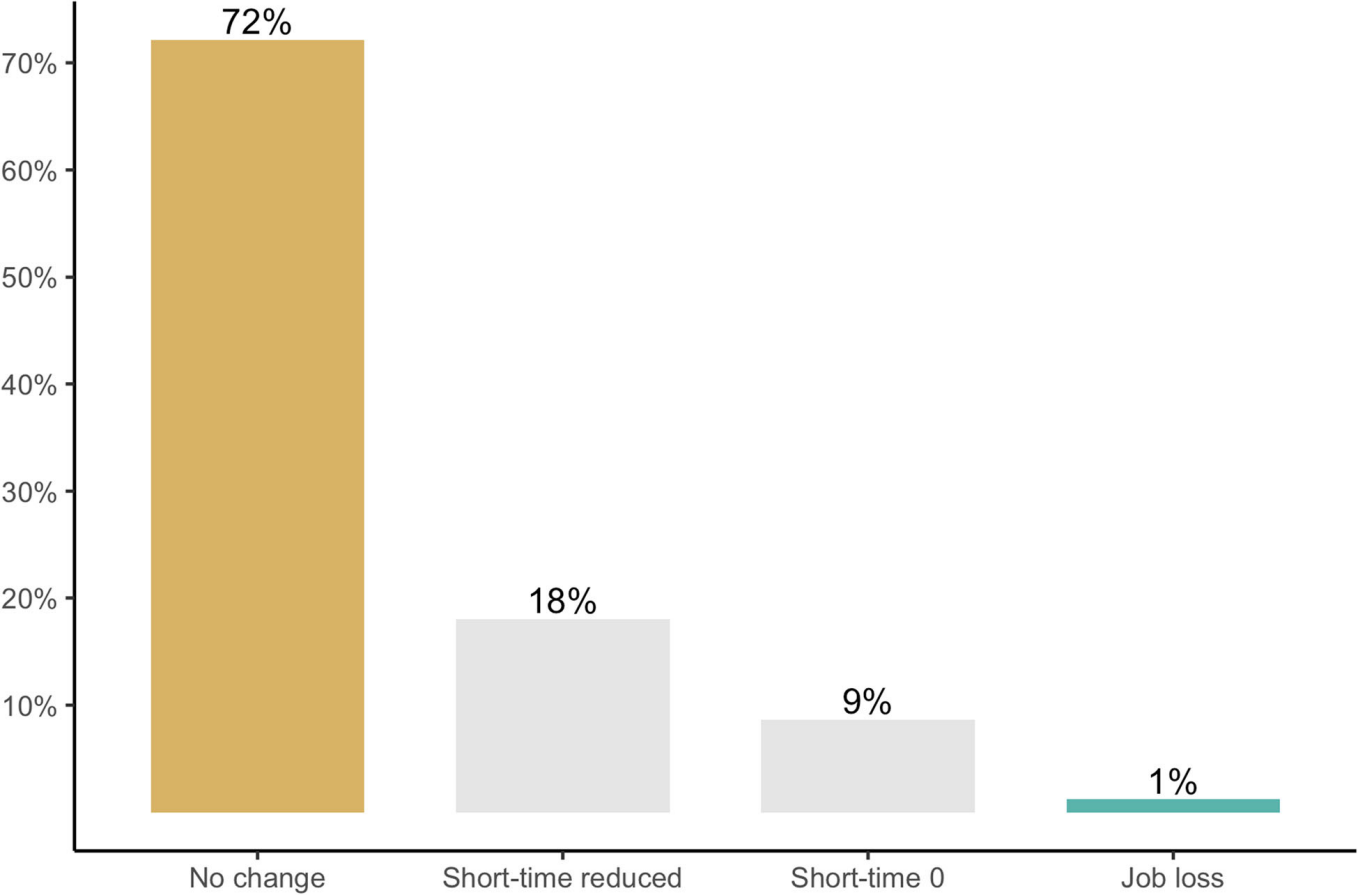
Self-reported changes in contracted working hours. Note: Short-time reduced = work hours temporarily partly reduced by employer; Short time 0 = work hours temporarily reduced to 0 by employer

Figures 4 and 5 show self-reported changes with regard to contracted working hours and home-office. Twenty-eight percent of participants experienced a change in their employment contract, while 27% were affected by mandatory short-time work, 1% lost their job as a result of the COVID-19 crisis. Fifty-one percent reported to WFH and of those, 20% reported doing so for the first time.

### Factors associated with perceived impact on work life

Table 2 shows OR comparisons between different subgroups concerning their evaluation of the degree to which their work life had worsened or improved due to the COVID-19 crisis, assessed by two separate dependent variables. Regarding perceived negative impact on work life, change in employment contract demonstrated the highest OR of reporting a deterioration of work life. The association was particularly strong in participants who had their contract reduced to mandatory short-time work with 0 working hours (OR = 9.72) and in those who had lost their job (OR = 35.07). Further, participants who reported a change in their work time had a significantly higher OR of reporting a deterioration of work life (OR = 2.95; 2.06). Finally, changes in leisure time and increased caring duties were significantly associated with perceived deterioration of work life. This association was particularly strong for a decrease in leisure time (OR = 1.62) and an increase in caring duties (OR = 1.58).

**Table 2:** Associations between sociodemographic factors, changes in routines, and positive/negative impact on work/private life

	Work life worsened		Work life improved		Private life worsened		Private life improved	
	OR	95% CI	OR	95% CI	OR	95% CI	OR	95% CI
Gender								
Female (Ref.)								
Male	0.91	0.78–1.07	0.93	0.78–1.09	1.05	0.90–1.22	0.86	0.73–1.02
Nationality								
Germany (Ref.)								
Switzerland	0.87	0.72–1.05	1.15	0.95–1.40	0.86	0.71–1.03	1.13	0.93–1.37
Age								
18–30 (Ref.)								
31–40	0.93	0.69–1.26	1.04	0.76–1.41	0.76	0.56–1.02	0.93	0.69–1.27
41–50	1.08	0.80–1.45	0.96	0.71–1.30	0.77	0.57–1.03	0.88	0.65–1.20
51–60	1.23	0.92–1.64	0.71*	0.52–0.95	0.76	0.57–1.00	0.80	0.59–1.07
61–65	1.55*	1.09–2.2	0.51**	0.35–0.74	0.58**	0.41–0.82	0.77	0.53–1.10
Living situation								
Alone (Ref.)								
Fam./part./shared	0.89	0.74–1.07	1.08	0.9–1.31	0.41***	0.34–0.49	1.74***	1.44–2.11
Change to contract								
No change (Ref.)								
Short-time^a^	3.45***	2.66–4.47	0.68**	0.52–0.89	1.22	0.95–1.58	1.32*	1.01–1.72
Short-time (0)^b^	9.72***	6.85–13.86	0.53***	0.37–0.77	1.06	0.78–1.45	1.57**	1.13–2.18
Job loss	35.07***	14.89–90.03	0.40*	0.16–0.92	1.21	0.60–2.44	1.67	0.77–3.56
Home office								
None (Ref.)								
Experienced	1.18	0.98–1.41	2.59***	2.14–3.14	1.02	0.85–1.22	1.72***	1.43–2.08
New	1.21	0.98–1.50	2.77***	2.22–3.45	1.07	0.87–1.32	1.41**	1.14–1.76
Working time								
Unchanged (Ref.)								
Decreased	2.95***	2.33–3.74	0.61***	0.48–0.78	0.90	0.72–1.14	0.89	0.70–1.13
Increased	2.06***	1.55–2.73	1.32	0.99–1.75	1.13	0.86–1.49	1.08	0.81–1.44
Leisure time								
Unchanged (Ref.)								
Decreased	1.62***	1.29–2.03	0.96	0.75–1.21	2.62***	2.09–3.28	0.85	0.67–1.08
Increased	1.27*	1.04–1.56	1.91***	1.54–2.38	1.30*	1.06–1.59	2.25***	1.82–2.79
Caring duties								
Unchanged (Ref.)								
Decreased	1.13	0.80–1.57	1.11	0.78–1.57	1.62**	1.19–2.22	1.35	0.96–1.89
Increased	1.58***	1.27–1.97	0.96	0.77–1.21	1.39**	1.12–1.72	1.33*	1.07–1.67

Note: *N* = 2118

* *p* < 0.05, ** *p* < 0.01, *** *p* < 0.001

^a^Work hours partly reduced

^b^Work hours reduced to 0

Regarding perceived positive impact of COVID-19 on work life, WFH had the highest OR of reporting an improvement in work life. The association was particularly strong in those who had started to WFH for the first time (OR = 2.77). Increase in leisure time was also significantly associated with a positive impact on work life. Further, older employees in the 51–60 and 61–65 age groups had significantly lower odds of reporting a positive impact of COVID-19 on work life (OR = 0.71; 0.61), as well as short-time employees, in particular those with a contract reduced to 0 working hours (OR = 0.53), and those who reported a decrease in work time (OR = 0.61).

### Factors associated with perceived impact on private life

Table 2 further shows OR comparisons within different subgroups concerning their evaluation of the degree to which their private life had worsened or improved due to the COVID-19 crisis, assessed by two separate dependent variables. Regarding perceived negative impact on private life, the subgroup of participants living with a partner, family, or in a shared housing had significantly lower odds of reporting the deterioration of their private life compared to those living alone (OR = 0.41). The odds of reporting deterioration of private life were lower also for the 61–65 age group (OR = 0.58). Finally, changes in the quantity of leisure time and quantity of caring duties were associated with perceived deterioration of private life, and this association was particularly strong for a decrease in leisure time (OR = 2.62) and a decrease in caring duties (OR = 1.62).

Regarding perceived positive impact on private life, the strongest association was with an increase in leisure time (OR = 2.25), followed by living with a partner, family, or in a shared housing (OR = 1.74); WFH, particularly among those with prior WFH experience (OR = 1.72); and with an increase in caring duties (OR = 1.33). Short-time workers had significantly higher odds of reporting a positive impact on their private life compared to workers without any change, especially those with a contract reduced to 0 working hours (OR = 1.57).

### Association between the perceived impact, self-reported changes, mental well-being and self-rated health

Table 3 shows the results of the associations between perceived overall impact, the self-reported changes in work and private life routines, and relevant health outcomes in terms of MWB and SRH, controlled for various sociodemographic variables. Regarding the perceived overall impact, participants who (strongly) agreed that COVID-19 had worsened their work life reported significantly lower MWB (OR = 0.61) compared to those who (strongly) disagreed. In addition, participants who neither agreed nor disagreed that their work life had worsened reported lower MWB (OR = 0.71) compared to those who (strongly) disagreed. A strong negative association could also be seen regarding perceived negative impact on private life: participants who (strongly) agreed that their private life had worsened reported lower MWB (OR = 0.62) and SRH scores (OR = 0.67) compared to those who (strongly) disagreed. Both outcomes were also negatively associated with employees who neither agreed nor disagreed that their private life had worsened (OR = 0.80; 0.66) compared to those who (strongly) disagreed. Finally, participants who (strongly) agreed that their private life had improved as a result of the COVID-19 crisis had higher odds of reporting a higher MWB score (OR = 1.39) compared to those who (strongly) disagreed.

**Table 3:** Associations between perceived impact on work/private life, self-reported changes, MWB, and SRH

	Mental well-being		Self-rated health	
	OR	95% CI	OR	95% CI
Work life worsened				
Disagree (Ref.)				
Neither/nor	0.71**	0.58–0.88	0.91	0.73–1.14
Agree	0.61***	0.49–0.76	0.76*	0.60–0.97
Work life improved				
Disagree (Ref.)				
Neither/nor	0.89	0.71–1.12	0.84	0.66–1.08
Agree	1.07	0.82–1.41	0.94	0.70–1.26
Private life worsened				
Disagree (Ref.)				
Neither/nor	0.80*	0.65–0.99	0.66***	0.53–0.83
Agree	0.62***	0.51–0.75	0.67***	0.54–0.83
Private life improved				
Disagree (Ref.)				
Neither/nor	1.03	0.83–1.28	1.04	0.83–1.32
Agree	1.39**	1.08–1.80	1.10	0.83–1.46
Change to contract				
No change (Ref.)				
Short-time (red.)	0.95	0.73–1.24	0.90	0.68–1.19
Short-time (0)	0.57***	0.41–0.79	0.49***	0.35–0.70
Job loss	0.71	0.34–1.47	0.79	0.35–1.81
Home-office				
None (Ref.)				
Experienced	1.05	0.88–1.26	0.99	0.81–1.20
New	1.14	0.92–1.40	1.07	0.85–1.33
Working time				
Unchanged (Ref.)				
Decreased	1.14	0.90–1.44	1.02	0.79–1.32
Increased	1.20	0.91–1.59	1.17	0.87–1.58
Leisure time				
Unchanged (Ref.)				
Decreased	0.81	0.64–1.02	0.92	0.72–1.18
Increased	1.23*	1.01–1.51	1.45**	1.16–1.82
Caring duties				
Unchanged (Ref.)				
Decreased	1.06	0.77–1.47	0.82	0.58–1.17
Increased	1.00	0.80–1.24	1.06	0.84–1.33

Note: *N* = 2118

* *p* < 0.05, ** *p* < 0.01, *** *p* < 0.001

Controlled for gender, age, country, and living situation

Regarding the impact of the self-reported changes in work and private life routines, mandatory short-time workers with a contract reduced to 0 working hours reported significantly lower MWB (OR = 0.57) and SRH (OR = 0.49) compared to participants without any change in their employment contract. In contrast, an increase in leisure time was positively associated with both better MWB (OR = 1.23) and SRH (OR = 1.45).

## Discussion

The present study aimed to examine the impact of the COVID-19 crisis on employees’ work and private life and the consequences for MWB and SRH in German and Swiss employees. The first objective of the study was to assess the perceived impact and self-reported changes related to COVID-19. Although the research has thus far mostly emphasized the negative impact of the COVID-19 crisis [9–12, 36], our data show that more than 40% of participants perceived no negative changes and over 10% even positive shifts in both life domains. This can be partly explained by the experienced changes in daily routines: 28% of participants were affected by a change in their employment contract and 49% by changes in the quantity of work time, confirming almost identical findings for Germany in the Eurofound report [12]. Also, quantity of leisure time and of caring duties changed for 58 and 26% respectively. The finding that about half WFH at least part of their working time, and 20% for the first time is also in line with Eurofound’s data where 24% reported WFH for the first time [12]. Overall, the proportion of people affected by changes in work and private life is comparable but hardly exceeds 50%, similar to the proportion of participants who reported a deterioration in their work and private life.

The second objective was to explore the factors associated with perceived impact on work and private life. A change in contracted work hours (i.e., mandatory short-time work, job loss), and changes in work time were strongly associated with reporting deterioration of work life. Those affected by short-time work experienced a significant disruption in their work routine as well as fear of losing the job, factors associated with increased level of distress and low MWB [7]. In consequence, employees whose contract had been reduced or terminated due to the lockdown measures are particularly vulnerable to developing mental health problems [11, 13]. Further, an increase in caring duties, and, perhaps more surprisingly, increase and decrease in leisure time were strongly associated with perceived deterioration of work life. Such changes in private life routines may require efforts for readjustments that can interfere with work and work-life balance. These readjustments may be particularly difficult for older employees (i.e., age group 61–65) who were more likely to report deterioration of their work life. They may be particularly sensitive to changes in daily structure and less flexible in adapting to a new situation, such as mandatory WFH, less personal contact with colleagues, and an increase in the use of digital technology.

WFH was most strongly associated with perceived positive impact of the COVID-19 crisis on work life, particularly in those reporting WFH for the first time, supporting evidence from Ipsen and colleagues [26]. This positive impact of WFH may be explained by a reduction or absence of commute time, more job autonomy, more flexible workdays, and ultimately, extra time for leisure. In fact, increased leisure time was another important factor associated with perceived positive impact of the COVID-19 crisis on work life. More time for leisure may allow for better recovery from work and rebuilding of personal resources [37, 38], which can then help an individual deal with work demands. In contrast, a change in contracted working hours and a decrease in work time were negatively associated with perceived positive impact on work life. A reduction in work time may not only cause financial problems, but also reduces important daily routines and social interactions at work, and may trigger fear of losing one’s job. Again, older employees may struggle more with the new situation and may be less successful in transforming it to their benefit, explaining why the oldest age groups, 54–60 and 61–65 years, were less likely to report an improvement in their work life.

Regarding the perceived impact on private life, participants living alone were more likely to report a deterioration and less likely to report an improvement of their private life compared to those living with a partner, family, or in a shared housing. The COVID-19 lockdown substantially restricted possibilities for social interactions beyond one’s own household, particularly affecting people living alone. For individuals who live alone, this may lead to feelings of loneliness [12], which in turn, threatens their MWB [39], highlighting the importance of having opportunities for direct exchange in such a crisis situation. This could also explain that an increase in caring duties, allowing for more exchange with family members, was associated with perceived positive shifts in private life. Further, an increase in WFH showed to be beneficial also to the private life, particularly to those experienced in WFH who did not need to first establish their workspace and new routines. Increase in leisure time and, more surprisingly, mandatory short-time work were also associated with positive impact on private life, as employees can engage more freely in activities they value. Interestingly, participants over 60 years old were less likely to report a deterioration of their private life. Older employees may be less dependent on the number of social contacts beyond their household, and they may have more mature emotion regulation strategies than the younger generations [40]. Indeed, mental well-being of the German elderly population (65+) remained largely unaltered during the early COVID-19 lockdown [41].

Finally, our third objective was to investigate how the perceived overall impact and self-reported changes induced by the crisis were associated with MWB and SRH. Low SRH has been associated with increased odds of depression [27], displaying the relevance of SRH for psychologically demanding situations, such as the COVID-19 pandemic. Our results suggest a strong negative association between the perceived negative impact on work and private life, MWB and SRH, indicating that this perception by itself is of relevance. It is of note that the perceived negative impact, particularly in private life, had such a strong association with SRH, which is more stable over time than MWB. In contrast, perceived positive impact on private life was associated with higher MWB. It seems that those who were able to cope with the COVID-19 crisis and translate the lockdown measures into some positive shifts in their private life, also benefited in terms of increased MWB.

Looking at the impact of the self-reported changes on MWB and SRH, mandatory short-time work with 0 contracted working hours was strongly associated with a lower MWB and SRH. Short-time work leads to significant losses of financial security and of daily structure and routines. Conversely, an increase in leisure time was positively associated with MWB, and the link was even stronger with SRH. More time for leisure gives extra opportunities for individuals to engage in meaningful activities that provide them with important resources that benefit their MWB and SRH. The overall strength of the associations indicates that MBW may be more affected by the perceived impact, as both are cognitive-emotional domains and are more dependent on the cognitive appraisal of one’s situation and emotional experience. SRH, on the other hand, may be more affected by actual changes in work and private life that increase or decrease opportunities to engage in activities that are perceived as beneficial to health.

### Limitations and strengths

A major limitation is the cross-sectional design, which allowed only to infer associations between variables but did not provide evidence of the directions of the associations or potential causality. Furthermore, the online survey created timely data on the immediate impact of the COVID-19 crisis situation. However, the self-reported data may be influenced by common method biases [42], such as social desirability bias [43] or self-selection bias, posing potential threats to the validity of our findings. Thus, we hired a professional panel data service that guarantees collection of high quality data. Moreover, we implemented various strategies in the questionnaire such as using disqualifying items to prevent invalid answers. The sociodemographic characteristics of our sample indicate a good representation of the target population. Finally, we did not control for all variables that might have affected the results. For instance, coping with a crisis and MWB differ individually and may be influenced by variables such as personality traits, resilience, or coping style [44–47]. However, our study aimed to provide a broad picture of both the negative and positive impacts of the COVID-19 crisis on a large, diverse sample of the working population. Thus, it was beyond the scope of this study to investigate individual differences and characteristics. In addition, a more complete, lengthy survey would have likely reduced the participation rate.

A strength of the present study is the relatively large and heterogeneous sample size that allowed us to conduct a detailed analysis and explore different subgroups within the sample. Another strength is the time point of the data collection launched at the beginning of April 2020, close to the first peak of the COVID-19 outbreak in Germany and Switzerland and onset of the related lockdown measures. This enabled us to capture a valid picture of the immediate impact of the lockdown measures. Moreover, the survey assessed the present situation, adding to the validity compared to a retrospective survey design. Finally, the combination of a subjective evaluation of the impact of the crisis with relevant, standardized public health indicators of MWB and SRH increases the relevance of the results to public health research and for policymaking.

## Conclusion and policy recommendations

The present study contributes to our understanding of the impact of the COVID-19 crisis on work and private life. It provides evidence on the covariates of a more negative/positive perceived impact and on the associations with MWB and SRH in the German and Swiss working populations. Employees whose employment contract was affected by the crisis seem to have felt the greatest negative impact on their work life. This highlights the crucial role of (un−/under-)employment in a crisis, as employment is associated with several health-promoting factors that cannot be substituted in any other way [48]. Moreover, the private life of employees living alone has been affected most negatively due to social isolation. Thus, psychological first aid also accessible online should be established particularly for these vulnerable groups [49]. Employers need to assure that they keep close social ties with and emotionally support employees with reduced contract or working hours. Moreover, rapid financial aids are needed to those who have lost their income partially or completely.

Nevertheless, we should also foster positive consequences of the crisis. In general, it seems that an increase in WFH was positive for work life. Learning from the beneficial effects of WFH in a crisis can inform future organizational and legislative policies to support this form of working. As employees experienced with WFH had a stronger positive impact on private life than first-timers, future WFH policies should include offering training and exchange of experience between employees on how to establish positive routines compatible with their private life. This will help employees to proactively identify their preferences and craft their work environment accordingly [50]. Further, an increase in leisure time was particularly positive for private life. More leisure time allows for dedicating extra time to activities one enjoys, and this may be beneficial also for recovery and detachment from work [51] and for mental health in general [52]. Thus, employees could also be trained in optimal crafting of their leisure time to strengthen these beneficial effects [53, 54].

Finally, we saw that besides the reported actual changes in work and private life, also the perception of the overall positive or negative impact is related to the health outcomes. This suggests to offer positive psychology trainings to employees helping them to purposefully focus on and make use of potential positive consequences of the crisis [55–57]. From a longitudinal research perspective, it would be interesting to further examine how the actual and perceived impact of the ongoing crisis as well as the associated health outcomes change over time and whether some of the new routines developed during the pandemic will be maintained in the long term.

To conclude, our study adds to recent evidence [58] that the Covid-19 crisis and related lockdown measures do not have solely negative impact. Rather, it affects vulnerable groups of individuals who need targeted support, while the majority of the population remain healthy or even experience positive shifts in their daily life.

## Supplementary Information

**Additional file 1.**

**Additional file 2.**

## Data Availability

The datasets used and/or analysed during the current study are available from the corresponding author on reasonable request. The R code used for the statistical analysis is available in the GitHub repository: https://github.com/jesuismartin/covid

## Abbreviations

MWB: Mental well-being
SRH: Self-rated health
WHO: World Health Organization
PHEIC: Public Health Emergency of International Concern
WFH: Work from home
EU: European Union
OR: Odds ratio
CI: Confidence interval

## Footnotes

1 Education estimates are based on data from *n* = 1194 participants who took part in a subsequent wave of data collection (December 2020), missing values (*n* = 924) were imputed using *mice* R package (for details see supplementary material). Education was not included in the regression models as the imputed data could potentially threaten the validity of our conclusions.

2 Short-time work is defined as “public programs that allow firms experiencing economic difficulties to temporarily reduce the hours worked while providing their employees with income support from the State for the hours not worked” (European Commission, 2020, Retrieved from: https://eur-lex.europa.eu/legal-content/EN/TXT/?qid=1587138033761&uri=CELEX%3A52020PC0139).

3 *None* = 0% WFH before COVID-19, 0% after; *Experienced* = at least 10% WFH before and at least 10% after COVID-19; *New* = 0% WFH before and at least 10% after COVID-19.

4 Participants were grouped into three categories according to their answers: *disagree* (1 + 2), *neither/nor* (3), *agree* (4 + 5).
