# Supplementary material for: Impact of the COVID-19 crisis on work and private life, mental well-being and self-rated health in German and Swiss employees: a cross-sectional online survey
Source: BMC Public Health. 2021 Apr 17;21:741. doi: 10.1186/s12889-021-10788-8 (PMC8052554; doi:10.1186/s12889-021-10788-8)
Supplement: Supplementary file 2 — Additional file 2. [file 12889_2021_10788_MOESM2_ESM.pdf]

Impact of the COVID-19 crisis on work and private life, mental well-being and self-rated health in German and Swiss employees: a cross-sectional online survey

Authors: Martin Tušl[a], Rebecca Brauchli[a], Philipp Kerksieck[a], Georg Friedrich Bauer[a]

[a]Public and Organizational Health, Epidemiology, Biostatistics and Prevention Institute, University of Zurich, Hirschengraben 84 CH-8001 Zurich, Switzerland

Survey

Dear participant,

For years, we have been studying the issue of reconciling work and family/private life. Especially now - in the current Corona crisis triggered by the COVID-19 pandemic - it is particularly important to find a way to reconcile work and family/private life in relation to our environment. We would be very pleased if you would support us with your answers to find clues for a healthy coping with the crisis.

Instructions for completing the questionnaire:

- Answer the questions spontaneously, as it applies to you at the moment.
- There are no right or wrong answers. It is exclusively about your personal opinion and experience.

Anonymity

- All information will be treated strictly confidentially. The data will be analyzed at the University of Zurich.
- The results will be reported completely anonymously, i.e. only in the form of average values or percentages.

Thank you very much for your participation!

Are you employed?
( ) Yes
( ) No

If not:
Were you employed until April 2020?
( ) Yes
( ) No

How many hours per week do you work as part of your employment contract?
( ) 0-9 hours
( ) 10-19 hours
( ) 20-29 hours
( ) 30-39 hours
( ) 40-49 hours
( ) more than 49 hours

Year of birth
(select)

Your gender
( ) Male
( ) Female
( ) Other / Do not want to answer

Country you live in
( ) Germany
( ) Switzerland
( ) Other

What is your current living situation?
( ) I live alone

( ) I live with my partner / family
( ) I live in a shared household
( ) Other

How many persons that need your care live in your household (e.g. children, parents)?
( ) None
( )1
( )2
( )3
( )4
( )5
( ) 6 or more

The following questions deal directly with the current COVID-19 (Corona) pandemic and the consequences of the government regulations it has triggered (business closures, school closures, event bans, contact reduction in public spaces, etc.), as well as the gradual relaxations of these regulations that have occurred in recent weeks. Hereafter, we refer to this collectively as the Corona crisis. Please compare your current situation with the situation as it was before the government regulations.

The Corona crisis has worsened my work life:
(1) Strongly disagree (2) Disagree (3) Neither/Nor (4) Agree (5) Strongly agree

If Agree or Strongly agree:
What has worsened in your work life specifically?
(short open answer)

The Corona crisis has improved my work life:
(1) Strongly disagree (2) Disagree (3) Neither/Nor (4) Agree (5) Strongly agree

If Agree or Strongly agree:
What has improved in your work life specifically?
(short open answer)

The Corona crisis has worsened my private life:
(1) Strongly disagree (2) Disagree (3) Neither/Nor (4) Agree (5) Strongly agree

If Agree or Strongly agree:
What has worsened in your private life specifically?
(short open answer)

The Corona crisis has improved my private life:
(1) Strongly disagree (2) Disagree (3) Neither/Nor (4) Agree (5) Strongly agree

If Agree or Strongly agree:
What has improved in your private life specifically?
(short open answer)

Have you been affected by any of the following employment changes as a result of the Corona crisis?
( ) Short-time work (reduced contracted working hours)
( ) Short-time work (reduced contracted working hours to 0)
( ) Job loss
( ) No change

Are you afraid of losing your job due to the Corona crisis?

( ) Not at all
( ) Not much
( ) Yes, somewhat
( ) Yes, very much

Has the quantity of your weekly working time changed as a result of the Corona crisis?
( ) Strongly decreased
( ) Decreased
( ) Unchanged
( ) Increased
( ) Strongly increased

Has the quantity of your weekly free time changed as a result of the Corona crisis?
( ) Strongly decreased
( ) Decreased
( ) Unchanged
( ) Increased
( ) Strongly increased

Has the quantity of your weekly caring duties changed as a result of the Corona crisis?
( ) Strongly decreased
( ) Decreased
( ) Unchanged
( ) Increased
( ) Strongly increased

The share of my work that I perform in the home office was/is as follows:
before the Corona crisis (in percent) 0%-100%
since the Corona crisis (in percent) 0%-100%

Well-being
Below are some statements about feelings and thoughts. Please tick the box that best describes your experience of each over the last two weeks:
( ) Never, ( ) Rarely, ( ) Some of the time, ( ) Often, ( ) All the time
I've been feeling optimistic about the future
I've been feeling useful
I've been feeling relaxed
I've been dealing with problems well
I've been thinking clearly
I've been feeling close to other people
I've been able to make up my own mind about things

Self-Rated health
In general, how would you evaluate your health?
( ) Very bad
( ) Bad
( ) Moderate
( ) Good
( ) Very good
